# Curious Symptoms from Worms

**Published:** 1861-08

**Authors:** H. L. Wilson

**Affiliations:** Atlanta, Georgia


### ARTICLE III.

*Curious Symptoms from Worms.* By H. L. Wilson, M. D., of Atlanta, Georgia.

On the morning of the 20th June, 1861, Mr. M. came for me in great haste, stating that his son, George—a little boy of seven years—had, for two days past, been gradually swelling, until he had grown to be a moster. I accompanied

Mr. M. home, and upon my arrival, found the child's abdomen considerably ascitic, the distention continuing up under the axilla and over the clavicle, giving his neck quite a goitrous appearance. I proceeded to examine the abdomen for ascites, but instead of finding fluctuation, I found the parts very hard and inelastic. I next felt of his neck, but did not find a crepitant or crackling sound, thereby proving the existence of emphysema; nor did I find the doughey feeling, indicating an infiltration of serum; but, to my surprise somewhat, there was a sensation imparted to the hand very similar to that experienced from feeling a bag tightly filled with wool. The eyes, also, were very much swollen, giving the little fellow, all together, a most hideous appearance. He had had no fever at all, nor did his tongue indicate much internal derangement.

The only symptoms upon which I could form my diagnosis, were nasal irritation, and hacking cough. So, in the absence of any stronger indications, I ordered the child to take the following:

℞ Fluid Ext. Spigelia.
" " Senna *aa* ℥ i.
Oleum Chenipodium Gutta iii.

To be repeated every three hours, until copious evacuations were effected.

June 21.—I saw George again to-day, found the tumefaction lessened, and the child playful—evidently better. The mother informed me that he had evacuated several ascarides during the previous day. Seeing no good reason why the treatment should be changed, I told her to give him the anthelmintic drops during the day, with a warm bath at night.

June 22.—I find my little patient appearently relieved this morning, the swelling having entirely disappeared. His mother informed me that during the past twelve or fifteen hours, he had discharged a great quantity of worms, in lumps, perhaps some seventy-five or eighty, after which the swelling entirely subsided, leaving him perfectly well.

Now, how the existence of ascarides could have produced such a state of affairs in the region of the neck, is somewhat mysterious to me. These are the facts, however, I give them for what they are worth.

